# Urine neutrophil gelatinase–associated lipocalin predicts outcome and renal failure in open and endovascular thoracic abdominal aortic aneurysm surgery

**DOI:** 10.1038/s41598-018-31183-1

**Published:** 2018-08-23

**Authors:** A. Gombert, I. Prior, L. Martin, J. Grommes, M. E. Barbati, A. C. Foldenauer, G. Schälte, G. Marx, T. Schürholz, A. Greiner, M. J. Jacobs, J. Kalder

**Affiliations:** 1 European Vascular Center Aachen, University Hospital Aachen, RWTH Aachen University, Maastricht, Germany; 2 Department of Intensive Care and Intermediate Care, University Hospital Aachen, RWTH Aachen University, Maastricht, Germany; 3 Department of Medical Statistics, University Hospital Aachen, RWTH Aachen University, Maastricht, Germany; 4 Department of Anesthesiology, University Hospital Aachen, RWTH Aachen University, Maastricht, Germany; 5 Department of Anaesthesia and Intensive Care, University of Rostock, Rostock, Germany; 6 Department of Vascular Surgery, Charité University Hospital Berlin, Berlin, Germany

## Abstract

Urine neutrophil gelatinase–associated lipocalin (uNGAL) has been evaluated as a biomarker for AKI detection and adverse outcome in open and endovascular thoracoabdominal aortic aneurysm surgery. This observational, retrospective study included 52 patients. UNGAL was measured peri-operatively (48 h) and correlated with AKI requiring dialysis, tracheotomy and adverse outcome. Mean patients’ age was 64.5 years. A total of 26.9% (*n* = 14) developed AKI, and 21.1% (*n* = 11) required dialysis, tracheotomy rate was 19.2% (*n* = 10) and in-hospital mortality rate was 7.6% (*n* = 4). uNGAL levels were related to AKI requiring dialysis at ICU (*p* = 0.0002), need for tracheotomy at baseline and admission on ICU (*p* = 0.0222, *p* = 0.0028, respectively), as well as adverse discharge modality (*p* = 0.0051, *p* = 0.0048, respectively). Diagnostic quality was good for uNGAL levels at admission to ICU regarding AKI requiring dialysis (sensitivity: 81.8% [48.2–97.7]; specificity: 87.8% [73.8–95.9]; area under the curve (AUC): 0.874 [0.752–0.949]). The diagnostic quality of uNGAL was favorable for the prediction of tracheotomy (sensitivity: 70.0% [34.8–93.3]; specificity: 83.3% [68.6–93.0]; AUC: 0.807 [0.674–0.903]) and adverse discharge (sensitivity: 77.8% [40.0–97.2]; specificity: 83.7% [69.3–93.2]; AUC: 0.817 [0.685–0.910]). uNGAL may be valuable as an post-operative predictor of AKI and adverse outcome after open and endovascular TAAA repair.

## Introduction

Open and endovascular repair of thoracoabdominal aortic aneurysms (TAAAs) is associated with a high risk of complications such as acute kidney injury^1,2^. Moreover, postoperative AKI is related to increased mortality rates after open, endovascular and emergency aortic aneurysm repair^3–6^.

In daily clinical routine, AKI detection is based on urine volume and serum creatinine levels. With renal function limitation of more than 50%, increased serum creatinine is detectable, which may be associated with oliguria or polyuria, yet these levels are not specific for impaired renal function^2,7,8^. Early biomarkers for AKI detection in fields of cardiovascular surgery are not clinically established, and a late diagnosis resulting in delayed treatment may be a consequence^9–11^. Hence, a biomarker facilitating early diagnosis of AKI after TAAA repair would be useful in facilitating early therapeutic intervention and guiding pharmaceutical treatment^12^.

NGAL, a 25 kDa protein that binds covalently to neutrophil gelatinase, has been reported as a potential marker of angiogenesis and in particular as an early marker of AKI after cardiac and abdominal aortic surgery^13–16^. Levels of NGAL may be influenced by different factors such as systemic infection and inflammation^17^. Elevated levels have been identified after impairment of kidney function, and NGAL seems to have a protective effect on cells that may be related to its ability to scavenge iron and to induce cell growth^18–20^. In murine models, NGAL is the most rapidly induced protein of nephrotoxic and ischemic AKI and is detectable 3 h following an initial kidney injury^21,22^. Furthermore NGAL has been described as biomarker of adverse outcome in different cardiovascular settings^23,24^.

Here we investigated the intriguing potential of urine NGAL (uNGAL) as a marker of AKI and adverse outcome in the context of complex endovascular and open TAAA surgery.

## Results

### Patient demographics

Fifty-two patients, of whom 25% (*n* = 13) were women, were included between May 2014 and November 2015. Mean age was 64.5 ± 10.4 years (range, 43–85 years). Patients were treated for TAAA by open surgical 55.7% (*n* = 29) or endovascular 44.3% (*n* = 23) approach; 40.3% (*n* = 21) had type II TAAA, 4% (*n* = 2) type III, and 55.7% (*n* = 29) type IV (Table 1).

**Table 1:** Patient Characteristics According to favorable and adverse discharge.

Mean + SD or median + range	All patients (N = 52)	favorable discharge (normal ward) (N = 43 [82.7%])	Adverse discharge (weaning, death within 30 days after surgery) (N = 9 [17.3%])	p-value (discharge modality comparison)
**Patients characteristics and treatment**				
Age	64.5 ± 10.4 (43; 85)	64.14 ± 10.8 (43; 85)	66.22 ± 8.7 (52; 77)	P = 0.5902
Gender (male)	39 (75.0)	32 (74.4)	7 (77.8)	P = 1.0000 OR = 0.83 (0.07; 5.36)
Open surgery	29 (55.8)	23 (53.5)	6 (66.7)	P = 0.7124 OR = 0.57 (0.08; 3.16)
Endovascular surgery	23 (44.2)	20 (46.5)	3 (33.3)	P = 0.7124 OR = 1.74 (0.32; 12.03)
BMI	27.1 ± 3.9 (18.2; 37.5)	27.5 ± 4.0 (18.2; 37.5)	25.6 ± 3.5 (19.8; 32.1)	P = 0.2006
Smoker	22 (42.3)	19 (44.2)	3 (33.3)	P = 0.7167 OR = 1.58 (0.29; 11.00)
Diabetes	6 (11.54)	6 (14.0)	0	P = 0.5745 OR = Infty (0.32, Infty)
Chronic kidney disease	7 (13.5)	4 (9.3)	3 (33.3)	P = 0.0900 OR = 0.21 (0.03; 1.82)
Coronary heart disease	21 (40.4)	18 (41.9)	3 (33.3)	P = 0.7236 OR = 1.44 (0.26; 10.02)
Arterial hypertension	47 (90.4)	38 (88.4)	9 (100)	P = 0.5726 OR = 0.00 (0.00; 4.04)
**Operation characteristics**				
Operation time	401.3 ± 99.0 (195; 600) N = 51	388.0 ± 95.9 (195; 600)	472.5 ± 88.6 (330; 600) N = 8	P = 0.0250*
Total ventilation time	980 (Q1: 570; Q3: 1980) (Min: 275; Max: 53805) N = 50	840 (Q1: 525; Q3: 1410) (Min: 275; Max: 6660)	43320 (Q1: 21615; Q3: 48660) (Min: 15675; Max: 53805) N = 7	P = 0.0001*
In- hospital stay	21 (Q1: 11; Q3: 32) (Min: 6; Max: 119) N = 51	18 (Q1: 10; Q3: 28) (Min: 6; Max: 45)	60 (Q1: 41.5; Q3: 68.5) (Min: 31; Max: 119)	P < 0.0001*
Stay on ICU	3 (Q1: 1; Q3: 7) (Min: 0; Max: 42) N = 51	2 (Q1: 1; Q3: 5) (Min: 0; Max: 32)	21.5 (Q1: 18.5; Q3: 32.5) (Min: 7; Max: 42) N = 8	P < 0.0001*
**Complications and mortality**				
AKI	14 (26.2%)	7 (16.3)	7 (77.8)	P = 0.0007* OR = 18.0 (2.5; 196.0)
AKI req. temporary Dialysis	11 (21.2%)	4 (9.3)	7 (77.8)	P < 0.0001* OR = 0.029 (0.003; 0.245)
Pneumonia	10 (19.2)	1 (2.3)	9 (100)	P < 0.0001* OR = 0.00 (0.00; 0.03)
Tracheotomy	10 (19.2)	1 (2.3)	9 (100)	P < 0.0001* OR = 0.00 (0.00; 0.03)
Spinal cord ischemia	2 (3.8)	0	2 (22.2)	P = 0.0271* OR = 0.00 (0.00; 0.68)
Myocardial infarction	0	0	0	—
Sepsis	7 (13.4)	3 (6.9)	4 (44.4)	P = 0.0125* OR = 0.09 (0.01: 0.78)
Surgical revisions	6 (11.5)	3 (6.9)	3 (33.3)	P = 0.5678 OR = 0.15 (0.02; 1.45)
In – hospital Mortality	4 (7.6)	3 (6.9)	1 (11.1)	P = 0.5441 OR = 0.60 (0.04; 35.39)
Total mortality	5 (9.6)	3 (7.0)	2 (22.2)	P = 0.2023 OR = 0.26 (0.03; 3.80)
Hereof pneumonia	2 (3.8)	2 (4.6)	0	P = 1.0000 OR = Infty (0.06; Infty)
Hereof hemorraghic	1 (1.9)	0	1 (11.1)	P = 0.1731 OR = 0.00 (0.00; 3.98)
Hereof small intestine ischemia	1 (1.9)	0	1 (11.1)	P = 0.1731 OR = 0.00 (0.00; 3.98)
Hereof cerebral bleeding	1 (1.9)	1 (2.3)	0	P = 1.0000 OR = Infty (0.01; Infty)

Examination of different patient characteristics separated by the discharge modality “favorable” and “adverse.” If data were missing, the sample size included is reported for the corresponding parameter. All variables are described as absolute frequencies n (%), mean ± SD or median (Q1, Q3), and ranges. ORs with 95% CIs are reported for dichotomous variables together with the *p* value.

## Complications and mortality

A total of 26.9% (*n* = 14) of patients developed AKI, 21.1% (*n* = 11) required temporary dialysis treatment, and 5.7% (*n* = 3) needed permanent dialysis after discharge from the hospital. No occluded renal artery stents or bypasses could be observed. Of the overall group, 19.2% (10/52) developed pneumonia, 23% (*n* = 12) needed re-intubation, and 19.2% (*n* = 10) received a tracheotomy.

Of the 13.4% (*n* = 7) who developed sepsis, six cases were related to pneumonia and one case to small intestine ischemia following embolization during open type III repair. Two patients (3.8%) developed spinal cord ischemia, one after an endovascular type 2 TAAA repair and one patient after an open type 3 TAAA repair.

Six patients (11.5%) underwent surgical revisions: 5.7% (*n* = 3) because of access-related wound complications and 3.8% (*n* = 2) for hemothorax. One patient (2%) needed multiple revisions, including bowl resection, because of small intestine ischemia.

The in-hospital mortality rate was 7.6% (*n* = 4), and the total mortality rate during the follow-up (mean follow-up, 13.2 months (±5.3, [2–20 months]) was 9.6% (*n* = 5). Of the latter, there were two cases of pneumonic sepsis, one each of cerebral bleeding and small intestine ischemia associated with pancreas necrosis and peritonitis, and one thoracic aortic rupture at 19 post-operative weeks after type IV repair.

### Correlation of uNGAL and biomarkers, clinical scoring systems, and outcome parameters

We observed an increasing correlation between uNGAL and serum creatinine over time. Furthermore, a significant correlation between uNGAL and the APACHE-II score was observed for all time points (ƍ = 0.457 admission to ICU; ƍ = 0.364 24 h after admission to ICU; ƍ = 0.439 48 h after admission to ICU). Additionally, a significant correlation for all time points of uNGAL and urine output could be assessed (ƍ = −0.320 admission to ICU; ƍ = −0.349 24 h after admission to ICU; ƍ = −0.559 48 h after admission to ICU).

Looking at a correlation of non-repeated factors with uNGAL at ICU, a significant correlation was found for length of ICU stay (ƍ = 0.390; *p* = 0.0046), as well as for the duration of dialysis (uNGAL: ƍ = 0.543; *p* < 0.0001). The ventilation time and in-hospital stay showed low to moderate correlations with uNGAL but were not significant (uNGAL: ventilation, ƍ = 0.272; *p* = 0.0557; in-hospital stay: ƍ = 0.265; *p* = 0.0598) (Table 2).

**Table 2:** Correlation of uNGAL with different biomarkers and scoring systems.

	Serum creatinine	Urine creatinine	Urea	Lactate	Urine excretion	APACHE II	AST	ALT
***Baseline***								
uNGAL	−0.065	—	—	—	—	—	—	—
***Admission on ICU***								
uNGAL	0.216 (N = 50)	0.119 (N = 24)	0.108 (N = 50)	—	−0.320*	0.457* (N = 48)	−0.004 (N = 49)	−0.118 (N = 48)
***12*** ***h after ICU***								
uNGAL	—	−0.333 (N = 28)	—	—	—	—	—	—
***24*** ***h after admission on ICU***								
uNGAL	0.454*	−0.025 (N = 22)	0.364*	0.413* (N = 48)	−0.349* (N = 51)	0.364* (N = 50)	0.333*	0.301* (N = 50)
***48*** ***h after admission on ICU***								
uNGAL	0.608* (N = 43)	−0.277 (N = 19)	0.443* (N = 44)	0.254 (N = 37)	−0.559* (N = 38)	0.439* (N = 35)	0.538* (N = 39)	0.443* (N = 39)

Correlation of uNGAL with different biomarkers using a Spearman’s correlation at 5 time points. *p < 0.05. If less than the 52 patients were included in the analysis, the corresponding sample size is given below the correlation value (AST: aspartate aminotransferase; ALT: alanine aminotransferase).

### Correlation of uNGAL levels and AKI requiring dialysis

**Figure 1:**
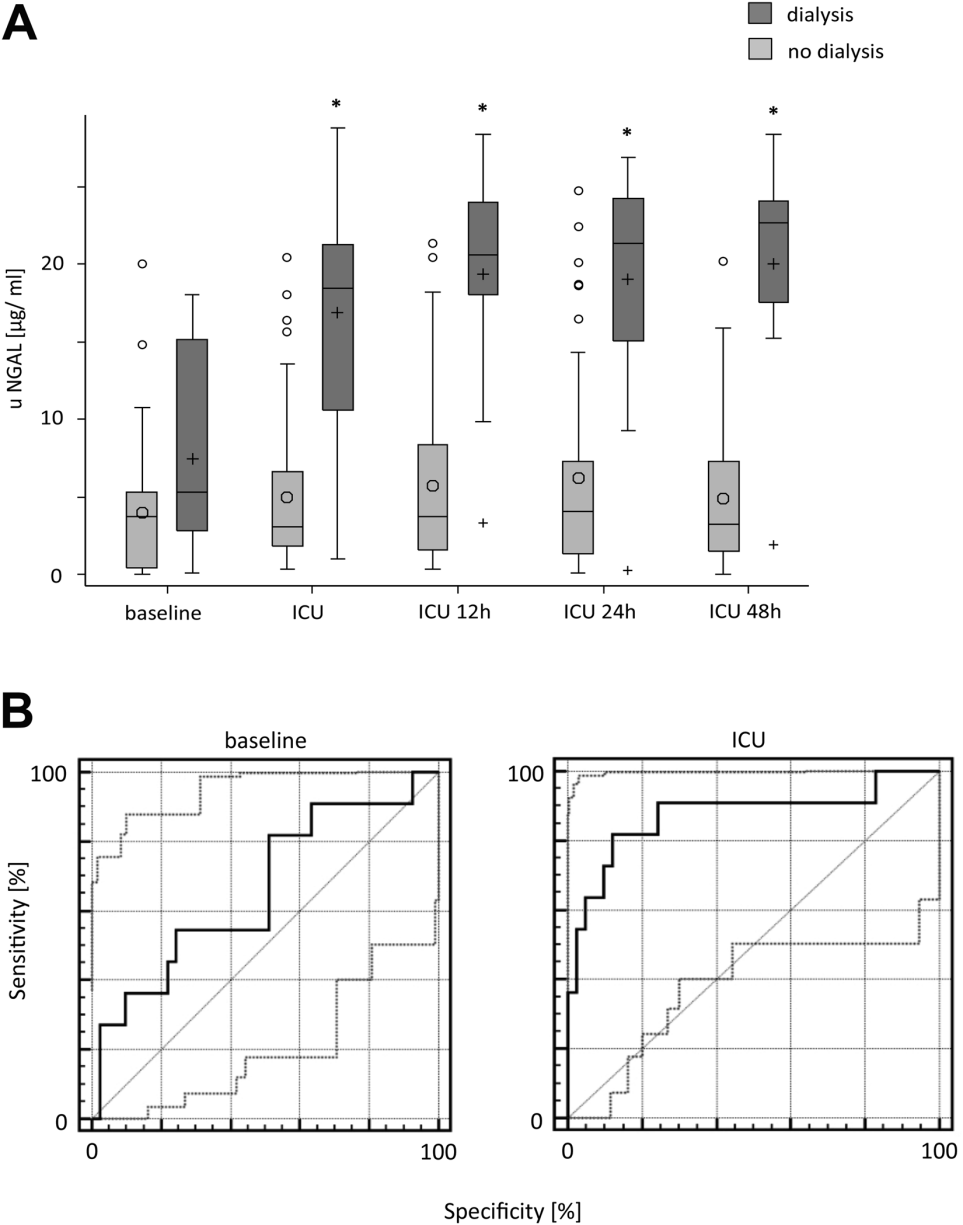
Relationship between uNGAL and patients suffering from AKI requiring dialysis analyzed for every time point (mean ± SD respectively median [Q1, Q3] if data were skewed. Tests: t-test or Wilcoxon rank-sum test. *p < 0.05. Baseline comparison corresponds to patients in need of dialysis. ROC analysis for uNGAL with respect to AKI and the need for dialysis treatment. 95% CIs of ROC curve indicated by dotted lines. For Se, Sp, and AUC, 95% CIs also are reported. *Good–moderate diagnostic quality: LR+ > 3; LR− < 0.3. **Excellent diagnostic quality: LR+ > 10; LR− > 0.1.

Starting with admission to ICU, uNGAL levels differed significantly among patients suffering from AKI requiring dialysis and those with non-impaired renal function. With the exception of baseline uNGAL levels, each time point showed a significant correlation. A ROC analysis for uNGAL with respect to AKI requiring dialysis treatment showed a good diagnostic quality for the time points ‘baseline’ and ‘ICU’ (baseline: Se = 81.8% [48.2–97.7], Sp = 48.8% [32.9–64.9], AUC = 0.661 [0.516–0.786], respectively; ICU: Se = 81.8% [48.2–97.7], Sp = 87.8% [73.8–95.9], AUC = 0.874 [0.752–0.949]). An uNGAL cut-off of 10.43 ng/ml for the time point ‘admission to ICU’ had the best predictive power for the development of an AKI requiring dialysis, with an AUC of 0.874. Furthermore, Wilcoxon tests revealed a significant difference of uNGAL between patients with and without AKI requiring dialysis for all time points except baseline (details in Fig. 1 and Supplemental Tables 1–2).

### Correlation of uNGAL-levels and need for a tracheotomy

**Figure 2:**
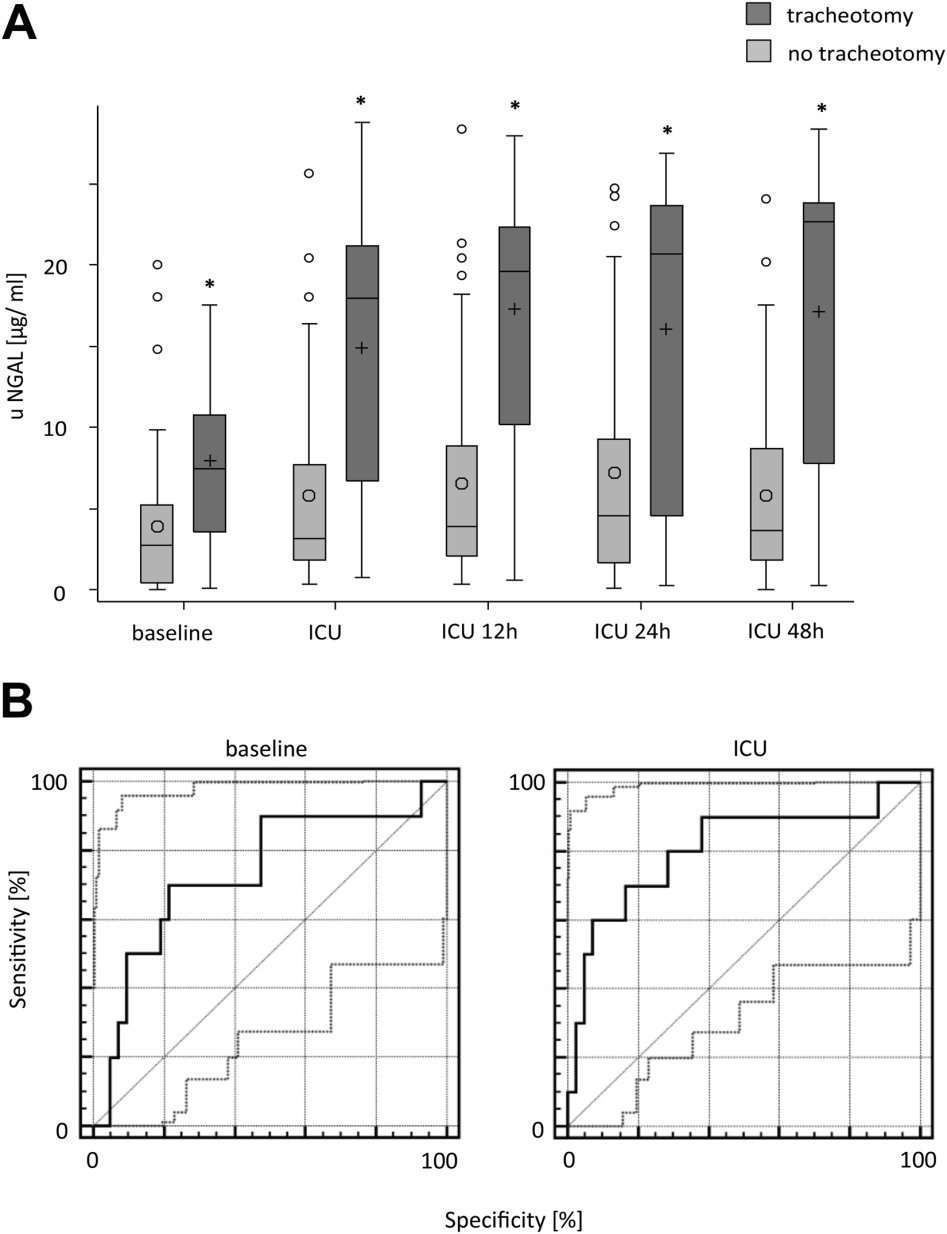
Relationship between uNGAL and tracheotomy dialysis analyzed for every time point (mean ± SD respectively median [Q1, Q3] if data were skewed. Tests: t-tests or the Wilcoxon rank-sum test. *p < 0.05. Baseline comparison corresponds to patients in need of dialysis. ROC analysis for uNGAL with respect to AKI and the need for dialysis treatment. 95% CIs of ROC curve indicated by dotted lines. For Se, Sp, and AUC, 95% CIs also are reported. *Good–moderate diagnostic quality: LR+ > 3; LR− < 0.3. **Excellent diagnostic quality: LR+ > 10; LR− > 0.1.

Considering the need for a tracheotomy, uNGAL levels differed significantly between the tracheotomy and non-tracheotomy patients for each time point. A ROC analysis for uNGAL with respect to needing a tracheotomy showed a good diagnostic quality for the time points ‘baseline’ and ‘ICU’ (baseline: Se = 70.0% [34.8–93.3], Sp = 78.6% [63.2–89.7], AUC = 0.736 [0.595–0.848], respectively; ICU: Se = 70.0% [34.8–93.3], Sp = 83.3% [68.6–93.0], AUC = 0.807 [0.674–0.903]). Similar to AKI, uNGAL cut-offs of 5.27 ng/ml and 10.43 ng/ml for the time points ‘baseline’ and ‘admission to ICU’ had the best predictive power for the need for a tracheotomy. Additionally, when we compared uNGAL between patients with and without tracheotomy, we observed a significant difference at all time points, including at baseline (details in Fig. 2 and Supplemental Tables 3–4).

### Correlation of uNGAL levels and adverse discharge modality

**Figure 3:**
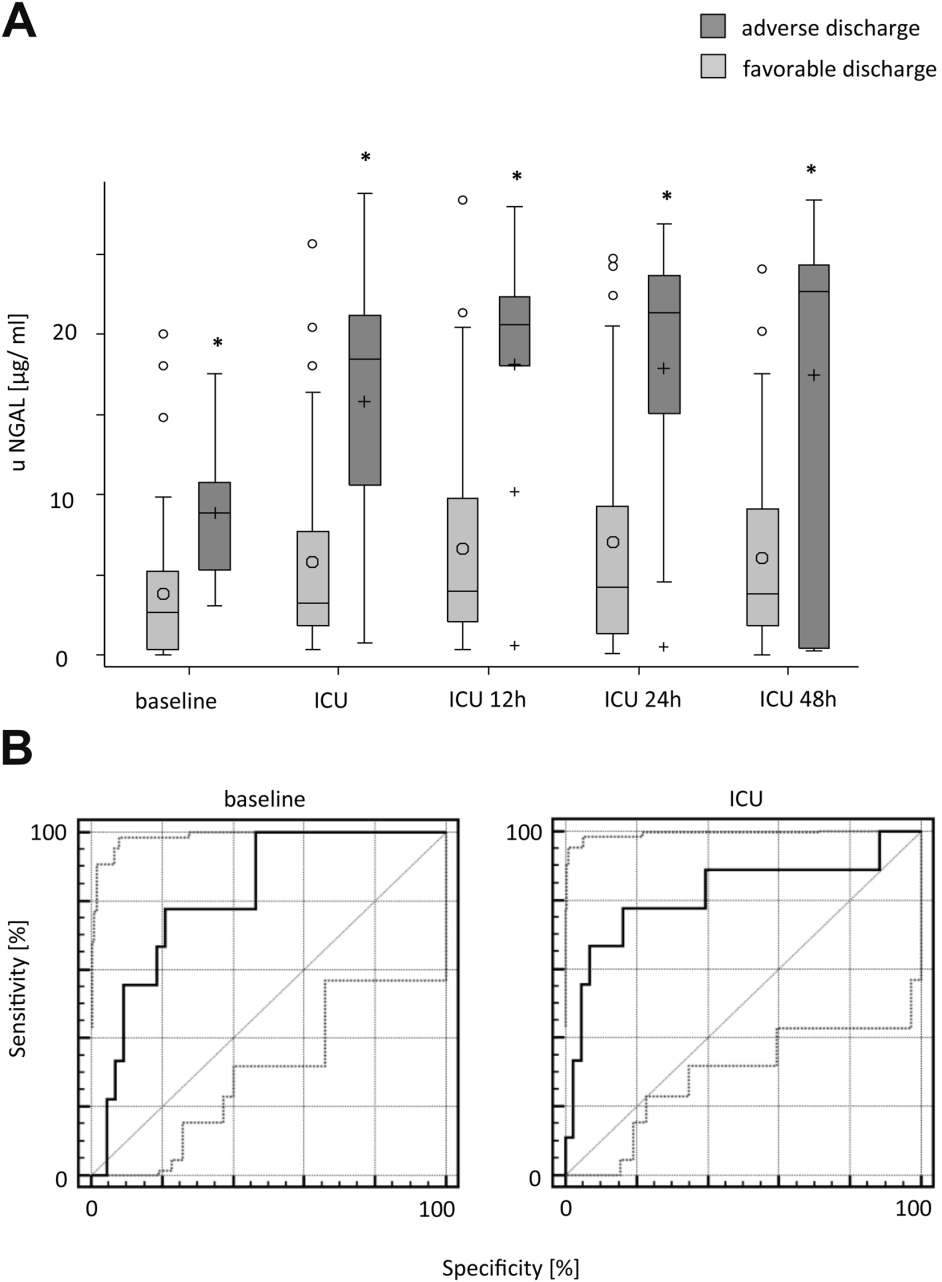
Relationship between uNGAL and adverse discharge modality (weaning ward or death) analyzed for every time point (mean ± SD respectively median [Q1, Q3] if data were skewed. Tests: t-tests or Wilcoxon rank-sum test. *p < 0.05. Baseline comparison corresponds to patients in need of dialysis. ROC analysis for uNGAL with respect to AKI and the need for dialysis treatment. 95% CIs of ROC curve indicated by dotted lines. For Se, Sp, and AUC, 95% CIs also are reported. *Good–moderate diagnostic quality: LR+ > 3; LR− < 0.3. **Excellent diagnostic quality: LR+ > 10; LR− > 0.1.

ROC analysis revealed that uNGAL predicted an adverse discharge modality, namely discharge via weaning or death, for each time point. With regard to the uNGAL measurement after 48 h, a good diagnostic quality could be assessed (Se = 71.4% [29.0–96.3], Sp = 97.6% [87.1–99.9]). Overall, the preoperative uNGAL levels had good predictive quality with an AUC value of 0.814. At the baseline measurement, a cut-off of 5.27 ng/ml (LR+ = 3.72 > 3 and LR− = 0.28 < 0.3) showed the best predictive power for an adverse discharge modality. Similarly, the cut-off value of >10.43 ng/ml after admission to ICU showed almost identical diagnostic quality with an increased specificity of 83.7%. A specificity of 97.6% 48 h after admission to ICU could be observed, resulting in a positive likelihood ratio of 29.29 > 10. Investigating uNGAL at each time point, we identified a significant difference between patients with a favorable and an adverse discharge modality at all time points (details in Fig. 3 and Supplemental Table 5–6).

### Coincidence of AKI requiring dialysis, tracheotomy, and discharge modality

The probability of an adverse discharge in dependence of a tracheotomy and AKI requiring dialysis was assessed to evaluate treatment dependencies in association with patient outcomes. We observed a complete or quasi-complete separation of the patients (*p* = 0.0242, *p* = 0.0012, Table 3), i.e., an adverse discharge modality was observed only if a tracheotomy was performed. Only one patient who had a tracheotomy had a favorable outcome, and this patient also had suffered from AKI requiring dialysis. Of those patients with adverse outcome, two died without requiring dialysis, and their uNGAL levels decreased within 48 h (details in Table 3 and Supplemental Table 7).

**Table 3:** Association between tracheotomy and discharge modality with regard to patients suffering from AKI with and without required dialysis.

Discharge modality depending on tracheotomy and AKI req. dialysis							
		AKI req. dialysis and			No AKI and		
		Tracheotomy			Tracheotomy		
		Yes	No	Total	Yes	No	Total
discharge mortality	favorable	1	3	4	0	39	39
	adverse	7	0	7	2	0	2
	Total	8	3	11	2	39	41
p (Fisher Test)		P = 0.0242*			P = 0.0012*		

Data listed in each of the combination cell corresponds to absolute frequency. *p < 0.05.

### Repeated measures analysis of uNGAL

In a longitudinal analysis, a significant impact of the baseline value of uNGAL on the post-surgical measured levels of uNGAL within the first 48 h after admission to ICU (Table 4; base: *p* = 0.0011) was observed. Overall, uNGAL did not change significantly over time (base: *p* = 0.6935). The repeated factors urea, urine, and serum creatinine were also identified as significantly related to uNGAL (Table 4; urea: *p* = 0.0462; urine: *p* = 0.0044; serum creatinine: *p* = 0.0018). Nevertheless, the slope estimate of urine was close to zero.

**Table 4:** Repeated measures analysis of uNGAL in a single parameter and Multivariable analysis of the longitudinal model.

Linear mixed model for log(uNGAL)					
Covariables	Estimate	SEM (Estimate)	DF resp Num DF/Den DF	t- resp. F-value	p-value
Base model					
Intercept	0.9606	0.2228	71.5	4.31	<0.0001*
Baseline (uNGAL)	0.1031	0.0297	50.1	3.47	0.0011*
Time Point (overall)			3/113	0.48	0.6935
ICU (Reference)	0	.	.	.	.
12 h after ICU	0.1154	0.1487	127	0.78	0.4390
24 h after ICU	−0.0152	0.1496	118	−0.10	0.9192
48 h after ICU	−0.0608	0.1568	122	−0.39	0.6989
**Single parameter analysis of the longitudinal model**					
Continuous covariables					
Age	0.01583	0.01435	48.7	1.10	0.2754
BMI	−0.06411	0.03861	48.8	−1.66	0.1032
Operation time	0.00291	0.00149	47.6	1.95	0.0573
Total ventilation time	0.00002	0.00001	47.8	1.99	0.0525
Serum creatinine (repeated)	0.4636	0.1451	112	3.20	0.0018*
Urea	0.01383	0.00686	116	2.02	0.0462*
Urine	−0.00024	0.00008	114	−2.90	0.0044*
Categorial covariable					
Gender (Male)	0.7529	0.3445	48.9	−2.19	0.0337*
Diabetes	0.3283	0.4682	48.6	0.70	0.4865
Stroke	0.1274	0.4453	48.3	0.29	0.7761
Endovascular procedure	0.2969	0.2974	48.9	1.00	0.3230
Chronic kidney disease	0.5158	0.4384	49.3	1.18	0.2450
Arterial hypertension	0.1481	0.5105	48.7	0.29	0.7730
**Multivariable analysis of longitudinal model**					
Intercept	−0.0086	0.3750	60.6	−0.02	0.9819
Baseline (uNGAL)	0.1045	0.0300	52.5	3.48	0.0010*
Serum Creatinine	0.3548	0.1440	108	2.46	0.0153*
Gender (Male)	0.7149	0.3324	48.4	−2.15	0.0365*
Time Point (overall)			2/64.3	0.15	0.8628
ICU (Reference)	0	.	.	.	.
24 h after ICU	−0.03958	0.1920	52	−0.21	0.8375
48 h after ICU	−0.09956	0.1850	76.6	−0.54	0.5920

Target variable: Log-transformed uNGAL values from admission to ICU (reference category of time measurement). The base model was included in all analyses subsequently. Test parameters of the base model are reported only for the plain base model and in the multivariable case, not for the single parameter (‘univariate’) analysis. In the multivariable model, the time point 12 h after ICU could not be considered because serum creatinine was not measured at this time point. *p < 0.05.

Analyzing patients and surgery characteristics in the single parameter model, sex influenced uNGAL levels significantly (*p* = 0.0337) while age did not show a significant effect (*p* = 0.2754). Judging from the estimates, the highest regression slopes were related to the sex (0.7529 ± 0.3445), chronic kidney disease (CKD) (0.5158 ± 0.4384), and serum creatinine levels (0.4636 ± 0.1451). Although the CKD impact was not significant, it had a large impact on data variation. Furthermore, the multivariable model confirmed a significant influence of baseline uNGAL value, serum creatinine, and sex on uNGAL (baseline uNGAL: *p* = 0.0010; serum creatinine: *p* = 0.0153; sex: *p* = 0.0365), but uNGAL levels still did not differ significantly over time (time: *p* = 0.8628). For the multivariable model, the largest slope estimate still corresponded to sex (0.7149 ± 0.3324) and serum creatinine (0.3548 ± 0.1440).

Looking at the estimate for sex, men showed increased uNGAL values in contrast to women. Nevertheless, discharge modalities did not differ significantly between women and men (*p* = 1.0000; Table 1).

## Discussion

Based on the results of this study, we could confirm that post-operatively measured uNGAL could be used as a postoperative biomarker for AKI requiring dialysis and as a predictive biomarker for needing tracheotomy and for adverse discharge modality (namely discharge from weaning ward or death) after open and endovascular TAAA repair. Furthermore, pre-interventional measured uNGAL levels correlated significantly with the need for tracheotomy and an adverse discharge modality. To our knowledge, no study to date has evaluated uNGAL as a biomarker for patient outcome and prolonged ventilation after cardiovascular surgery.

NGAL has been successfully used in different settings for the diagnosis and prognosis of AKI. Although several studies have investigated the value of NGAL to predict AKI in pediatrics, nephrology, and heart surgery, only limited evidence exists for its predictive abilities after TAAA surgery^25–28^. Chang *et al*. described the use of new biomarkers such as NGAL for AKI detection after endovascular aortic repair^29^. Kalimeris *et al*. emphasized the value of elevated NGAL levels as predictors of AKI after repair of abdominal aortic aneurysm^30^. These findings support our results on the predictive ability of uNGAL baseline measurement.

Comparing the value of serum NGAL (sNGAL) and uNGAL in the aortic aneurysm setting, Kokot *et al*. identified an increased predictive value for uNGAL compared with sNGAL regarding the probability of AKI^31^. In the present study, we observed a significant correlation of uNGAL with serum creatinine levels and a significant negative correlation with urine volume. These findings underline a direct relation of uNGAL and impaired renal function after TAAA repair. Of interest, two of the patients with elevated uNGAL levels who died post-operatively did not develop AKI. In both cases, uNGAL dropped within 48 h to a normal level. The pre-operative elevation of uNGAL in these patients may be a consequence of an assumed function of NGAL as a stress response protein, indicating a reduced general condition, which could influence postoperative outcomes^20^. Furthermore the course of uNGAL in these cases may correlate with a reported decreased specificity of uNGAL as biomarker for AKI^32^. Only a study including a larger cohort of patients favorably in a multicentric setting could answer this question appropriately.

The function and meaning of NGAL as a stress response protein cannot clearly be discriminated in the setting of this study from its relevance as an early biomarker of AKI. It would have been favorable to correlate the course of uNGAL with further biomarkers of the innate immune response such as Interleukin 6 (IL-6), which has been described as potential predictor of the post-interventional inflammation status after major surgery^33^. Yet, the survey of IL-6 during the first days on ICU was fragmentary, so a potential correlation between uNGAL and IL-6 could not be assessed in the reported setting. In this context, Abella *et al*. described a regulatory role for NGAL in the innate immune response. NGAL is involved in a multitude of physiological and pathophysiological processes, such as apoptosis, infection and inflammation^34^. According to Kjeldsen *et al*., NGAL secretion by neutrophils, induced by tumor necrosis factor (TNF) and lipopolysaccharide (LPS), is activated by inflammation and infection^35^. Thus elevated baseline levels of NGAL as in the current study may be related to an altered immune status of the patients, which may be in turn related to adverse outcome^36^. Accordingly, the results regarding the predictive role of baseline uNGAL in this study may be consistent with the results of Lindberg *et al*., who described Plasma-NGAL as independent predictor of all-cause mortality and major adverse cardiovascular event in general population^37^. Finally, this thesis could not be validated by further findings in our study.

Nevertheless, while an increasing correlation between uNGAL and serum creatinine levels as well as urinary extraction could be assessed within the first 48 h after open and endovascular TAAA surgery, the initial assumption of uNGAL as a predictor of AKI still stands.

The multivariate analysis of uNGAL highlighted the influence of the baseline level of uNGAL and serum creatinine with post-surgical measurements of uNGAL, which strengthens the predictive power of uNGAL regarding postoperative AKI with the need for dialysis. With regard to the sex-specific differences of uNGAL, our results support the findings of Thrailkill *et al*., who identified significantly increased uNGAL levels in male patients with diabetes^38^. These results together may indicate a different applicability of uNGAL for men and women. Even if the outcome for men and women had not differed significantly in the present study, this finding seems important regarding a potential implementation of uNGAL as a urinary biomarker.

uNGAL levels and adverse discharge correlated significantly, and every patient who died or was discharged via the weaning ward showed already elevated uNGAL levels at baseline. Furthermore, the trend increased after admission to ICU. In agreement, Siew *et al*. found uNGAL as a prognostic biomarker for outcome of patients suffering from AKI^39^. The observed correlation of elevated uNGAL levels with the APACHE II score also underscores its ability to predict patient outcome after complex aortic surgery, and an association of post-surgical AKI and the APACHE II score has been described before^40,41^. Post-operative AKI after complex aortic surgery is directly related to adverse outcome, so the consistent results regarding the predictive power of uNGAL seem conclusive^4,40^.

Tracheotomy was conducted in this study if a prolonged artificial respiration was required. With regard to the correlation of elevated uNGAL levels and tracheotomy or adverse discharge, an almost complete concordance was found. Except for one patient who suffered from post-operative AKI and tracheotomy but was not discharged via the weaning ward, all patients who received a tracheotomy had an adverse discharge modality. In agreement, other authors also have described a correlation of tracheotomy with postoperative AKI and adverse outcome^42,43^.

In terms of the elevated NGAL-levels and their correlation with patients’ outcome, an early detection of elevated NGAL-levels by use of rapid testing kit may enable early medical and interventional treatment options such as dialysis therapy^44^.

Certain limitations of the present study must be taken into account while evaluating the discussed results: A prospective, multicenter study including more homogenous patients treated by open or endovascular means would have improved the quality of the assessed data. Otherwise, TAAA is a rare disease, and few vascular surgery centers perform open and endovascular treatment regularly. A discrimination of the function of NGAL as a stress response protein vs. its function as a kidney injury marker would be helpful. Currently, no panel of biomarkers associated with inflammation or kidney injury is available, which would enable a clear separation of uNGAL as a biomarker for inflammation or AKI in the described setting. The assessment of further biomarkers for the immune response was not possible during this study, hence a potential correlation of uNGAL and those biomarkers was not conducted. An exclusion of patients with pre-existing chronic renal failure would be useful regarding the homogeneity of this patient cohort. Still, although no significant influence regarding pre-existing CKD could be observed, the presented findings emphasize the relevance of uNGAL as a postoperative biomarker of AKI and predictor of adverse outcomes after open and endovascular TAAA repair. Although our analysis was conducted in a hypothesis-generating manner, the results are concordant with what has been reported in the literature.

## Methods

### Ethics approval and consent to participate

The local ethics committee approved this study (University Hospital Aachen EK004/14). This study was performed in accordance with the Declaration of Helsinki in its actual form. Written informed consent was obtained preoperatively from all subjects.

### Inclusion and exclusion criteria

Patients suffering from TAAA larger than 6 cm, defined according to the Crawford classification, were eligible for inclusion^45^. Exclusion criteria were age below 18 years, pregnancy, chronic kidney disease requiring permanent dialysis treatment. No emergency procedures were included.

### Clinical and laboratory data collection

In this retrospective study, data on demographics, medical history, and admission diagnosis as well as daily physiological variables, surgical interventions, need for dialysis, and any kind of adverse event were collected from medical records and electronic bedside flow charts (IntelliSpace Critical Care and Anesthesia; Philips Healthcare, Andover, Massachusetts, USA). During the surgical treatment, according to standardized and consistent operations, blood and urine samples were collected from patients at five predefined time intervals: pre-interventionally, at admission to the intensive care unit (ICU), and at 12 h, 24 h, and 48 h after admission to ICU. AKI was defined according to the Kidney Disease Improving Global Outcomes (KDIGO) criteria^46^. In this study the serum creatinine criteria were used to define AKI, whereat urine volume was not applied for AKI determination. Baseline creatinine was the lowest pre-intervention value. AKI was defined by a reduction in kidney function with an increase in serum creatinine (>26.4 µmol/L) or percentage increase of serum creatinine above 50%, as recommended in current guidelines^47^. In this pilot study we focused on AKI requiring dialysis.

### Biomarkers

Plasma and urine samples were incubated (3 G and 4 °C) for 10 min. The samples were stored at −20 °C for less than 2 weeks and afterwards stored at −80 °C until further processing by enzyme-linked immunosorbent assay (ELISA), performed according to the manufacturer’s instructions (Human Lipocalin 2/NGAL ELISA BioVendor, Brno, Czech Republic). The ELISA kits were of the highest reagent grade and used without further purification. Laboratory investigations were blinded to the clinical status of the patients throughout the study. Following the recommendations of the manufacturer, the normal and pathological reference ranges were established based on the control sample in our laboratory. Based on the results of a previous study, we focused on the assessment of uNGAL.

### Surgical protocol

#### Open surgery

The operative protocol for open TAAA repair has been published in detail before^48,49^. Briefly, it includes intubation with a double lumen endotracheal tube, cerebrospinal fluid drainage, perioperative monitoring of Motor Evoked potentials (MEP), sequential aortic clamping if possible, extracorporeal circulation with distal aortic perfusion, selective visceral perfusion and mild hypothermia of 32 °C to 33 °C^50^. Custodiol^®^ (Dr. Franz Köhler Chemie, Austria) at 44 °C was used for renal perfusion instead of blood perfusion. This approach had been described to protect the kidneys from ischemic organ damage^51^. Thoracolaparotomy through the fifth to eighth intercostal spaces depending on the extent of the aneurysm was used for surgical access as well as a groin cut-down to the femoral vessels for placement of the extracorporeal circulation cannulas.

#### Endovascular surgery

The procedure was performed under general anesthesia, and the same protocol was used for the neurological monitoring. The detailed procedure of fenestrated endovascular aortic aneurysm repair has been described before^52^. In cases of endovascular procedure, renal perfusion was not interfered with directly. Radiation and iodinated contrast solution were used conservatively to reduce the toxic effects on the kidneys. Furthermore, a reduced contrast solution dose (one fourth of the standard dose) for the selective angiography of the renal arteries was used because it has been described as protective regarding acute kidney failure^53^.

### Endpoints

Levels of uNGAL and their correlation with AKI following open and endovascular aortic repair was the primary endpoint. Correlations of uNGAL levels and tracheotomy as well as adverse discharge modality were assessed as secondary endpoints as these dichotomous events are related to poor outcome. Furthermore, correlations with different parameters and scoring systems such as APACHE II and Lactate have been observed.

Tracheotomy was indicated in case of extended artificial ventilation. In our hospital, a tracheotomy is performed after a minimum 96 h of ventilation. Adverse discharge modality was defined as discharge via weaning ward or death. As no patient was discharged from ICU, this modality of discharge was not defined as adverse discharge modality. All patients who survived the first 30 days were contacted between December 2015 and January 2016.

### Statistical analyses

For description, all continuous variables are expressed as mean values ± standard deviation (SD) or 95% confidence intervals (CIs). For heavily skewed distributions, the median, the 0.25–0.50 quantile (Q1), and the 0.75 quantile (Q3) were used instead. Categorical variables are expressed as absolute frequencies and percentages. Correlations between continuous parameters were assessed by the Spearman correlation coefficient for each time point (“rho”, ƍ). Some measurements in the data are missing completely at random; a systematic bias could not be detected.

The uNGAL level distribution was skewed, so exact Wilcoxon signed-rank tests were chosen to compare uNGAL for different factors between single time points. Comparisons between frequencies were conducted using a χ² test or Fisher’s exact test in cases of small cell frequencies (≤5). For patient characteristics with respect to their discharge, exact odds ratios (ORs), corresponding 95% (penalized) likelihood Cis, and *p* values are given in Table 1.

Linear models with repeated measures were applied to evaluate the impact of certain metabolic factors on uNGAL. As a base model, we considered the fixed time effect (repeated factor), preoperative NGAL value (baseline value), and a random intercept. All further linear models are extensions of the base model. The response parameter uNGAL was log transformed to meet the model requirements. A Kenward–Rogers adjustment was used to account for the small sample size. For the covariance, an AR (1) structure was assumed. Model fit was evaluated with the help of residual plots.

Given the small sample size, we focused on analyzing the influence of single parameters (univariate analysis). Therefore, we considered the clinical factors of age and sex and all metabolic factors that showed interesting correlations or were considered relevant according to literature and experience, separately as additional covariables for the base model. Furthermore, we performed a multivariable analysis for uNGAL modeling in addition to the base model for the impact of sex, creatinine levels in the serum, and AKI/dialysis simultaneously because they were the most promising predictors within the univariate analysis. Considering these parameters simultaneously improved the model according to the AIC (Akaike’s information criterion) value. At first, tracheotomy also was considered, but because it worsened the model fit (AIC) and did not have a significant influence, we omitted it from the model. We report for all fixed-effect covariables the (slope) estimate, its standard error, degrees of freedom (DF), the value of the test statistic (t-value), and the *p* value. Because we considered more than two time points, the *p* value of the overall F-Test (type 3) is reported for the overall time effect together with the two DFs (Num DF/Den DF) and the value of the F-statistic (F-value). We assessed any effect in the statistical models as significant if the corresponding *p* value fell below the 5% margin. No alpha adjustment was carried out because this study was considered exploratory.

A receiver operating characteristic (ROC) analysis was performed to evaluate the diagnostic property of uNGAL with respect to each patient’s direct discharge category (favorable/adverse), tracheotomy, and AKI requiring dialysis. Sensitivity (Se), specificity (Sp), likelihood ratios (LR±), area under the curve (AUC), and the Youden-optimal cut-off (maximize Se + Sp − 1) are reported together with the plotted ROC curves.

Boxplots were chosen to present the data distribution of uNGAL for selected patient characteristics over time. Spaghetti plots were used to illustrate the concrete course of uNGAL over time for patients with adverse direct discharge.

Statistical analysis was performed using SAS for Windows, Version 9.4 (SAS Institute, Cary, NC, USA), and “Proc Mixed” was used for the repeated measure analysis. ROC analysis was performed using MedCalc for Windows, version 12.7.7.0 (MedCalc Software, Ostend, Belgium).

## Conclusion

uNGAL may be used as a postoperative biomarker of AKI requiring dialysis, tracheotomy and adverse discharge modality after open and endovascular TAAA repair.

## Electronic supplementary material

Title page

Supplementary Dataset 1

## Data Availability

The datasets used and analyzed during the current study are available from the corresponding author upon request.
