# Supplementary material for: Urine neutrophil gelatinase–associated lipocalin predicts outcome and renal failure in open and endovascular thoracic abdominal aortic aneurysm surgery
Source: Sci Rep. 2018 Aug 23;8:12676. doi: 10.1038/s41598-018-31183-1 (PMC6107559; doi:10.1038/s41598-018-31183-1)
Supplement: Supplementary file 1 — Title page [file 41598_2018_31183_MOESM1_ESM.docx]

**Urine neutrophil gelatinase–associated lipocalin predicts outcome and renal failure in open and endovascular thoracic abdominal aortic aneurysm surgery**

A. Gombert^1*+^, I. Prior^1+^,L. Martin^2^, J. Grommes^1^,M. Barbati^1^, A. Foldenauer^3^, G. Schälte^4^, G. Marx^2^, T. Schürholz^5^, A. Greiner^6^, M. J. Jacobs^1^, J. Kalder^1^

^1^European Vascular Center Aachen- Maastricht, University Hospital Aachen, RWTH Aachen University, Germany

^2^Department of Intensive Care and Intermediate Care, University Hospital Aachen, RWTH Aachen University, Germany

3 Department of Medical Statistics, University Hospital Aachen, RWTH Aachen University, Germany

4 Department of Anesthesiology, University Hospital Aachen, RWTH Aachen University, Germany

5 Department of Anaesthesia and Intensive Care, University of Rostock, Rostock, Germany

6 Department of Vascular Surgery, Charité University Hospital Berlin, Berlin, Germany

Correspondence to *agombert@ukaachen.de

^+^A.Gombert and I. Prior share first authorship.
