# Supplementary material for: Urine neutrophil gelatinase–associated lipocalin predicts outcome and renal failure in open and endovascular thoracic abdominal aortic aneurysm surgery
Source: Sci Rep. 2018 Aug 23;8:12676. doi: 10.1038/s41598-018-31183-1 (PMC6107559; doi:10.1038/s41598-018-31183-1)

**Urine neutrophil gelatinase–associated lipocalin predicts outcome and renal failure in open and endovascular thoracic abdominal aortic aneurysm surgery**

A. Gombert^1*+^, I. Prior^1+^,L. Martin^2^, J. Grommes^1^,M. Barbati^1^, A. Foldenauer^3^, G. Schälte^4^, G. Marx^2^, T. Schürholz^5^, A. Greiner^6^, M. J. Jacobs^1^, J. Kalder^1^

^1^European Vascular Center Aachen- Maastricht, University Hospital Aachen, RWTH Aachen University, Germany

^2^Department of Intensive Care and Intermediate Care, University Hospital Aachen, RWTH Aachen University, Germany

3 Department of Medical Statistics, University Hospital Aachen, RWTH Aachen University, Germany

4 Department of Anesthesiology, University Hospital Aachen, RWTH Aachen University, Germany

5 Department of Anaesthesia and Intensive Care, University of Rostock, Rostock, Germany

6 Department of Vascular Surgery, Charité University Hospital Berlin, Berlin, Germany

Correspondence to *agombert@ukaachen.de

^+^A.Gombert and I. Prior share first authorship.

Supplement

| **AKI req. dialysis** | | | |
| --- | --- | --- | --- |
|  | **Yes (n=11)** | **No (n=41)** | **p-value** |
| **uNGAL** | | | |
| Baseline* | 5.31 (2.85; 15.11) | 3.69 (0.43; 5.27) | 0.1067 |
| ICU | 18.41 (10.62; 21.28) | 3.05 (1.85; 6.65) | 0.0002* |
| 12h on ICU | 20.55 (18.02; 24.01) | 3.77 (1.58; 8.39) | < 0.0001* |
| 24h on ICU | 21.36 (15.08; 24.21) | 4.10 (1.33; 7.29) | 0.0003* |
| 48h on ICU | 22.67 (17.52; 24.07) | 3.27 (1.52; 7.32) | 0.0001* |

Supplement 1: Relationship between uNGAL and patients with AKI and dialysis analyzed for every time point (median [Q1, Q3], since data was skewed). Tests: Wilcoxon ranked sum test. * p <0.05. Baseline comparison corresponds to patients in need of dialysis.

| **Time Point** | **Youden** | | | | | **AUC** |
| --- | --- | --- | --- | --- | --- | --- |
|  | **Optimal- Cut-Off** | **Sensitivity [%]** | **Specificity [%]** | **LQ+** | **LQ-** |  |
| Baseline | >2.69 | 81.8 [48.2-97.7] | 48.8 [32.9-64.9] | 3.72* | 0.28* | 0.661 [0.516-0.786] |
| ICU | >10.43 | 81.8 [48.2-97.7] | 87.8 [73.8-95.9] | 6.71* | 0.21* | 0.874 [0.752-0.949] |

Supplement 2: For Se, Sp and AUC the 95% confidence intervals are reported additionally. *Good-moderate diagnostic quality: LQ+ >3; LQ- <0.3. **: Excellent diagnostic quality: LQ+ >10; LQ- >0.1.

| **Tracheotomy (at ICU)** | | | |
| --- | --- | --- | --- |
|  | **Yes (n=10)** | **No (n=42)** | **p-value** |
| **uNGAL** | | | |
| Baseline* | 7.43 (3.60; 10.76) | **2.77 (0.43; 5.21)** | 0.0222* |
| ICU | 17.93 (6.74; 21.14) | **3.14 (1.85; 7.70)** | 0.0028* |
| 12h on ICU | 19.62 (10.15; 22.31) | **3.86 (2.09; 8.84)** | 0.0014* |
| 24h on ICU | 20.69 (4.55; 23.67) | **4.56 (1.63; 9.24)** | 0.0399* |
| 48h on ICU | 22.66 (7.80; 23.82) | **3.66 (1.83; 8.69)** | 0.0320* |

Supplement 3: Correlation between uNGAL and tracheotomy analyzed for every time point (median [Q1, Q3], since data was skewed). Tests: Wilcoxon ranked sum test. * p <0.05. Baseline comparison corresponds to patients in need of tracheotomy.

| **Time Point** | **Youden** | | | | | **AUC** |
| --- | --- | --- | --- | --- | --- | --- |
|  | **Optimal- Cut-Off** | **Sensitivity [%]** | **Specificity [%]** | **LQ+** | **LQ-** |  |
| Baseline | >5.27 | 70.0 [34.8-93.3] | 78.6 [63.2-89.7] | 3.27* | 0.38 | 0.736 [0.595-0.848] |
| ICU | >10.43 | 70.0 [34.8-93.3] | 83.3 [68.6-93.0] | 4.20* | 0.36 | 0.807 [0.674-0.903] |

Supplement 4: For Se, Sp and AUC the 95% confidence intervals are reported additionally. *Good-moderate diagnostic quality: LQ+ >3; LQ- <0.3. **: Excellent diagnostic quality: LQ+ >10; LQ- >0.1.

| Direct discharge modalities | | | |
| --- | --- | --- | --- |
|  | favorable discharge (normal ward)  N=43 | Adverse discharge (weaning, death)  N=9 | p-Value |
| **uNGAL** | | | |
| Baseline* | 2.69 (0.38; 5.21) | 8.84 (5.31;10.76) | 0.0051* |
| ICU | 3.23 (1.85; 7.70) | 18.41 (10.62; 21.14) | 0.0048* |
| 12h on ICU | 3.95 (2.09; 9.79) | 20.55 (18.02; 22.31) | 0.0030* |
| 24h on ICU | 4.27 (1.33; 9.24) | 21.36 (15.08; 23.67) | 0.0092* |
| 48h on ICU | 3.81 (1.86; 9.11) | 22.67 (0.40; 24.31) | 0.0720* |

Supplement 5: uNGAL levels compared between discharge modalities analyzed for every time point (median [Q1, Q3], since data was skewed). Tests: Wilcoxon ranked sum test. * p <0.05.

| **Time Point** | **Youden** | | | | | **AUC** |
| --- | --- | --- | --- | --- | --- | --- |
|  | **Optimal- Cut-Off** | **Sensitivity [%]** | **Specificity [%]** | **LQ+** | **LQ-** |  |
| Baseline | >5.27 | 77.8 [40.0-97.2] | 79.1 [64.0-90.0] | 3.72* | 0.28* | 0.814 [0.682-0.908] |
| ICU | >10.43 | 77.8 [40.0-97.2] | 83.7 [69.3-93.2] | 4.18* | 0.27* | 0.817 [0.685-0.910] |
| 12h after ICU | >9.81 | 88.9 [51.8-99.8] | 79.1 [64.0-90.0] | 4.25* | 0.14* | 0.835 [0.706-0.923] |
| 24h after ICU | >14.29 | 77.8 [40.0-97.2] | 81.4 [66.6-91.6] | 4.18* | 0.27* | 0.791 [0.655-0.891] |
| 48h after ICU | >20.19 | 71.4 [29.0-96.3] | 97.6 [87.1-99.9] | 29.29** | 0.29* | 0.721 [0.573-0.841] |

Supplement 6: Roc-Analysis for uNGAL with respect to direct discharge. For Se, Sp and AUC with 95% confidence intervals are reported additionally. *Good-moderate diagnostic quality: LQ+ >3; LQ- <0.3. **: Excellent diagnostic quality: LQ+ >10; LQ- >0.


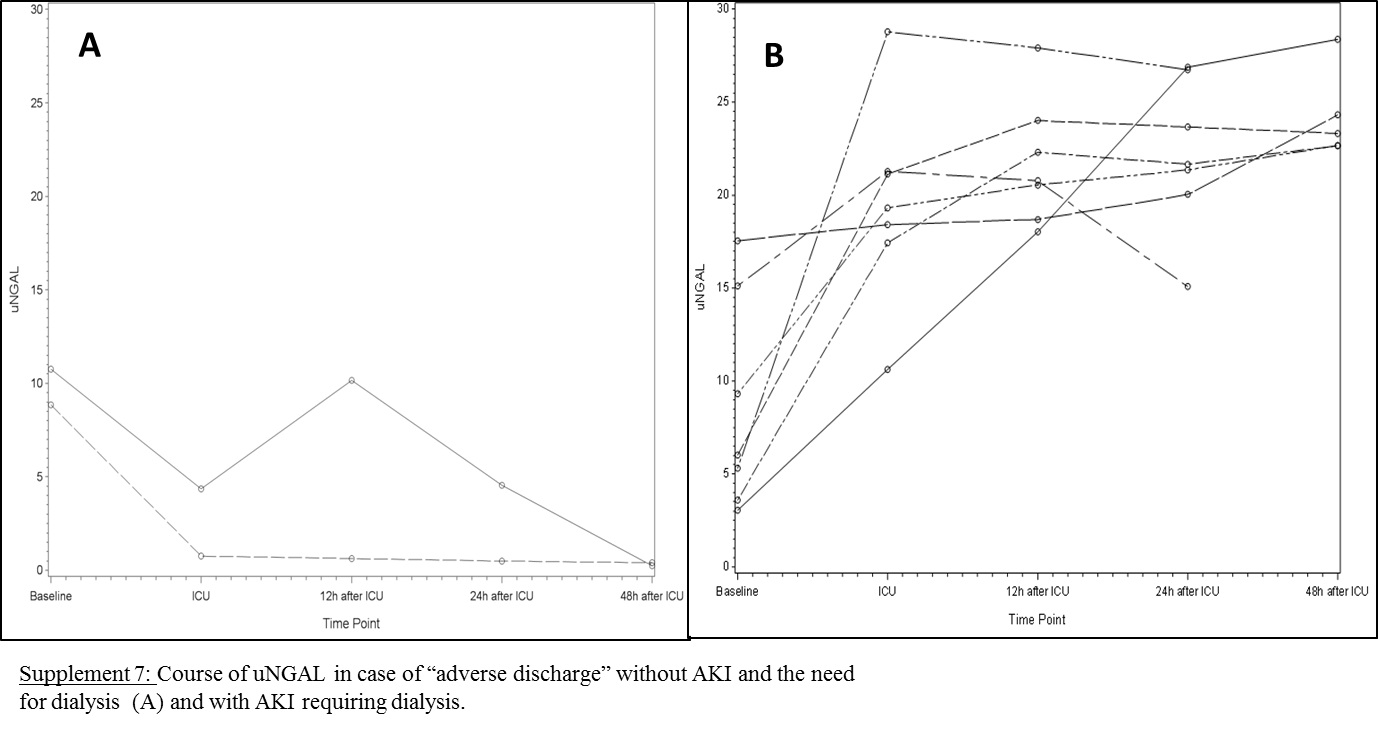

Supplement: Supplementary file 2 — Supplementary Dataset 1 [file 41598_2018_31183_MOESM2_ESM.docx]
